# Supplementary material for: Virus infection of the CNS disrupts the immune-neural-synaptic axis via induction of pleiotropic gene regulation of host responses
Source: eLife. 2021 Feb 18;10:e62273. doi: 10.7554/eLife.62273 (PMC7891934; doi:10.7554/eLife.62273)
Supplement: Supplementary file 8. [file elife-62273-supp8.docx]

Supplementary File 8

**
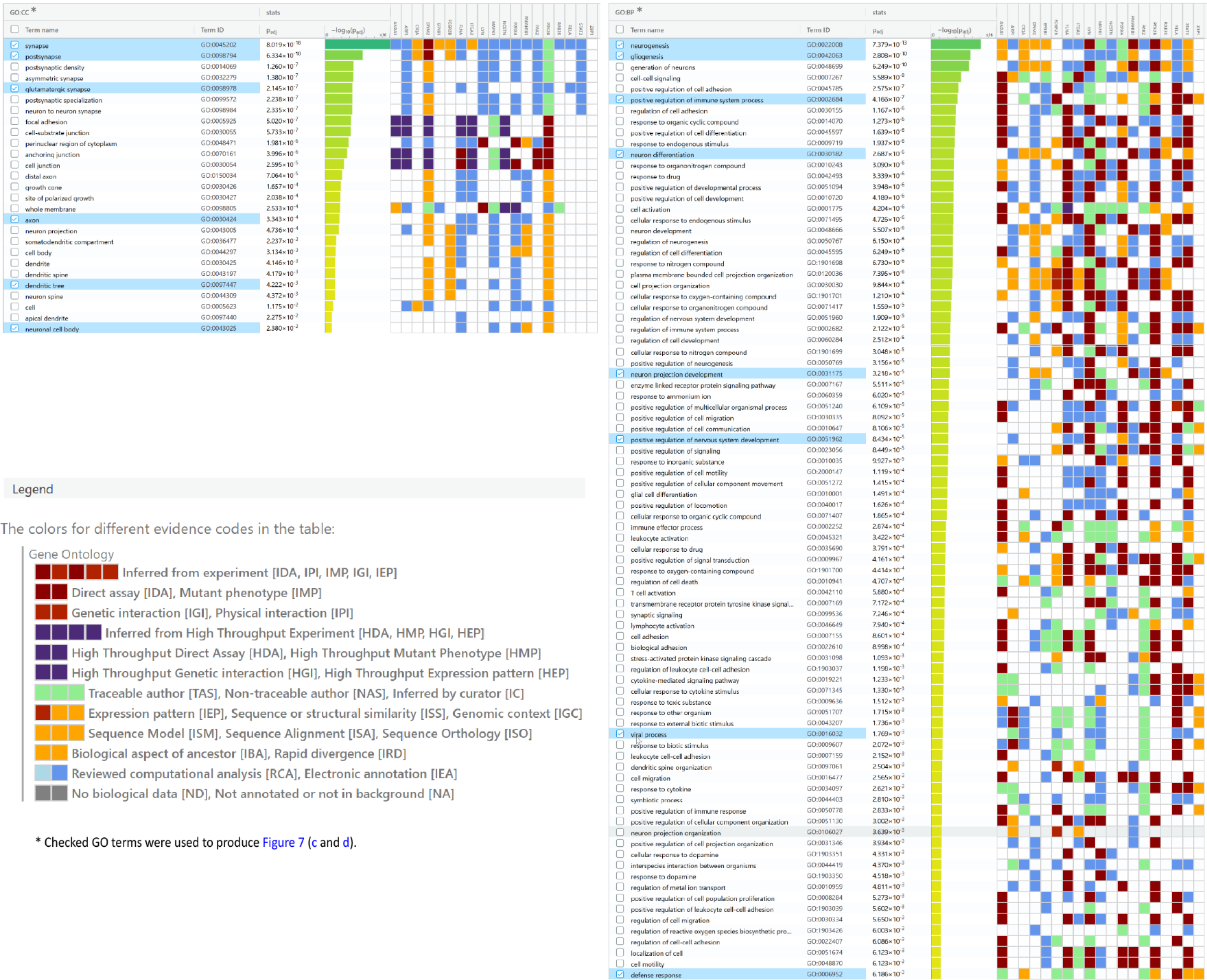
Upregulated immune-neural-synaptic pleiotropic DEGs: significantly enriched CC and BP GO terms**

**Downregulated immune-neural-synaptic pleiotropic DEGs: significantly enriched CC and BP GO terms**

**
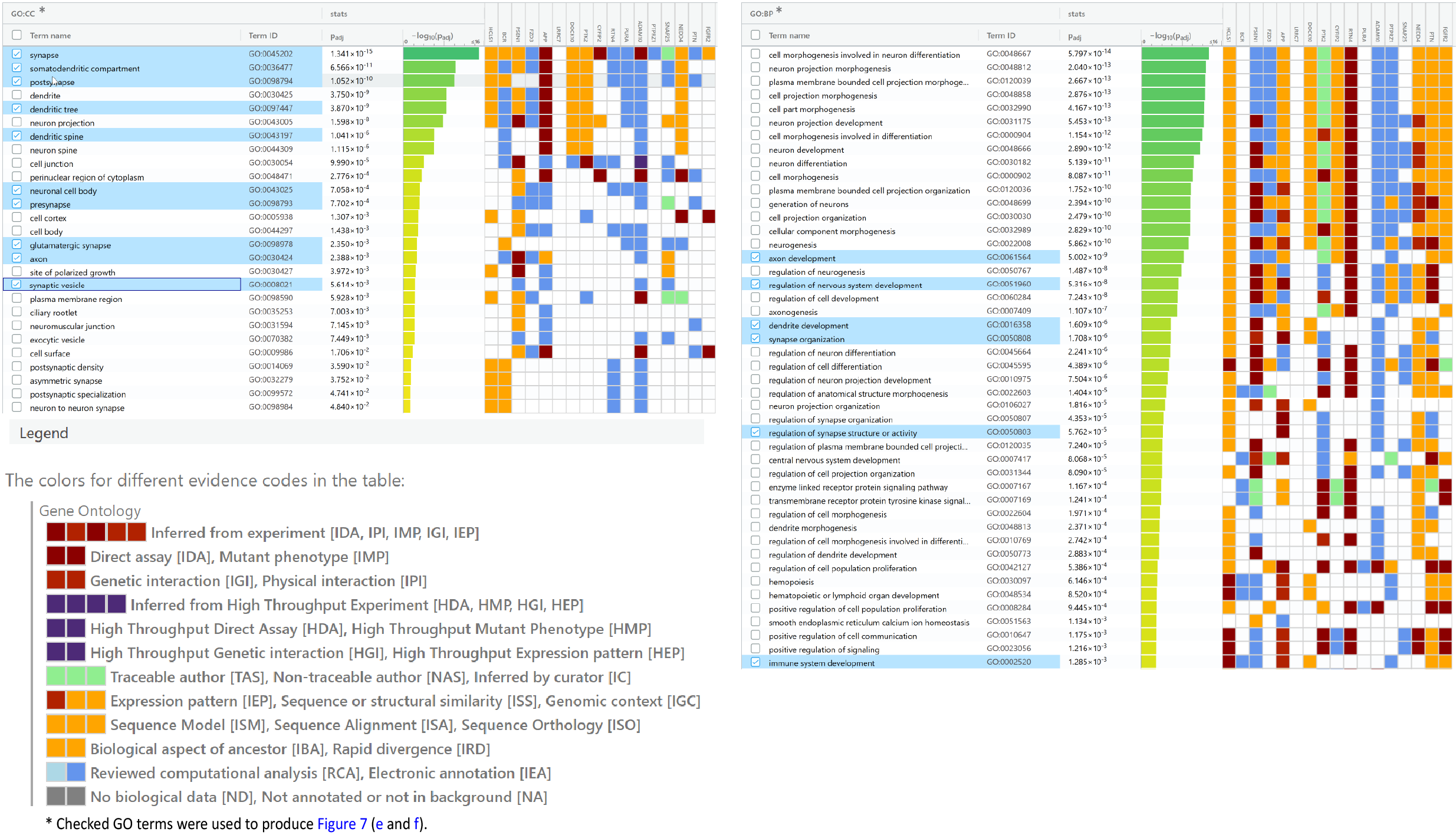
**
