# Supplementary material for: Virus infection of the CNS disrupts the immune-neural-synaptic axis via induction of pleiotropic gene regulation of host responses
Source: eLife. 2021 Feb 18;10:e62273. doi: 10.7554/eLife.62273 (PMC7891934; doi:10.7554/eLife.62273)
Supplement: Supplementary file 10. [file elife-62273-supp10.docx]

Supplementary File 10

**List of primers and probes used for qPCR validation of DNA microarray results**

| **Gene coding for** | **Type** | **5'-3' sequence** |
| --- | --- | --- |
| Calnexin | Forward | GTCAAGCCAGATGACTGGAT |
|  | Reverse | GGTACATACTCGGGCTCATCA |
|  | Probe | AAGATGCCCCTGCTAAGATTCCAGAT |
| TATA-box binding protein associated factor 11 | Forward | CTGACGGAGACGCAGATG |
|  | Reverse | GTCTTCCCTTTCAACTGTTGTTA |
|  | Probe | CCTCCGCTGCAGCTTCTTTCAAGT |
| amyloid beta A4 protein-like | Forward | AGTACCTACTGATGGCAATG |
|  | Reverse | TCATGTGCATGTTCAGTCT |
|  | Probe | CTGCTGGCTGAACCCCAG |
| beta-2-microglobulin | Forward | GCGCTACTCTCTCTTTCTG |
|  | Reverse | TTTCCATTCTCTGGTGGAT |
|  | Probe | ACTCCAAAGATTCAGGTTTACTCACG |
| Chemokine (C-X-C motif) ligand 10 | Forward | TGTTAATCCAAGGTCTTTAG |
|  | Reverse | TCTTCTCACCCTTCTTTTT |
|  | Probe | ATTATTCCTCCAAGTCAATTTTGTCC |
| tenascin C | Forward | ACGAGGTGTCCCTCATCTC |
|  | Reverse | AACACGTCGAAGGTTCCT |
|  | Probe | CGCAGAGGCGACATGTCAAGCAACC |
| glial fibrillary acidic protein | Forward | TTCCTCAAGAACCGGATCT |
|  | Reverse | CTGGCTGCCTATAGACAGAA |
|  | Probe | TCTCCAGATCCAGACGGGCC |
| complement component 3 | Forward | GACTTGATGATCTGCTCAAT |
|  | Reverse | GAGCCAGGAGTGGACTATT |
|  | Probe | TACTCGTCAAAGTCATTGGACAGC |
| gamma-aminobutyric acid (GABA) A receptor, alpha 2 | Forward | GAACTGGCCATCGAGCAGAT |
|  | Reverse | TTCAACCCTTTCGCATTGTC |
|  | Probe | CAACGAGTCACTCCTGCGCCCG |
| glutamate receptor, ionotropic, AMPA 1 | Forward | CATGCAGCAGTGGAAGAATAG |
|  | Reverse | TCACCTTCACCCCATCGTA |
|  | Probe | ACCACACTCGGGTGGACTGGAAAAGACCC |
| Calbindin 1 | Forward | TTATTCTTCTCGCACAGATC |
|  | Reverse | GAGTTCAATAAGGCTTTTGA |
|  | Probe | CATTTTCATCTATGTATCCATTGCCG |

**Validation of WNV-ND transcriptome**


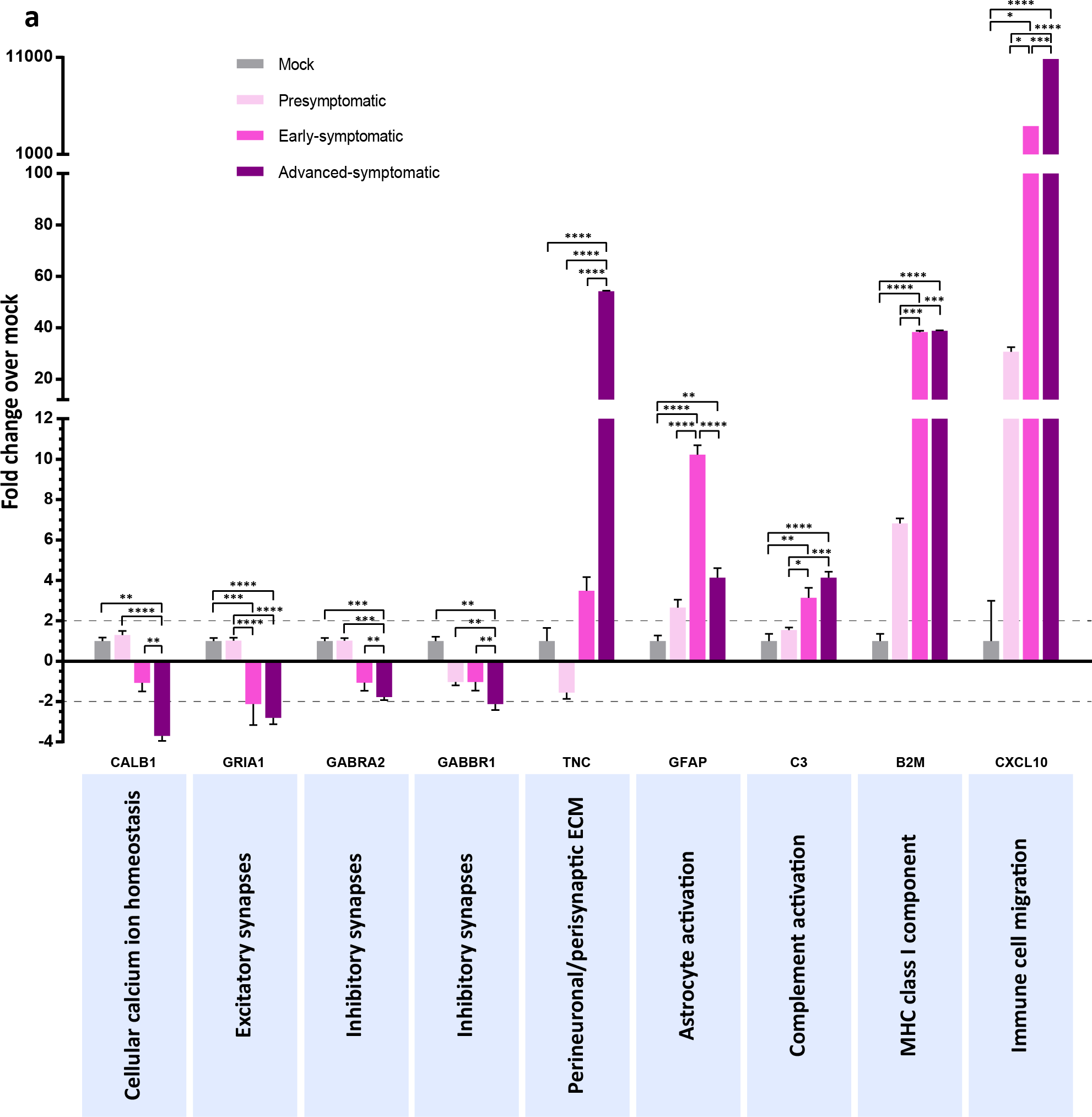


**Validation of WNV-ND transcriptome at the levels of RNA expression and affected biological processes.** (**a**) The levels of expression of select genes by qPCR in the cerebella of NHPs at the presymptomatic (3 dpi; n=3), early-symptomatic (7 dpi; n=3), and advanced-symptomatic (9 dpi; n=3) stages of WNV-ND relative to mock (n=3). Only statistically significant differences are indicated by brackets: *p<0.05, **p<0.01, ***p<0.001,

****p<0.0001 by one-way ANOVA. Error bars, STDEV. Dashed lines indicate the chosen fold change cutoff for the differentially expressed genes in the microarray data. Biological processes to which each select gene was mapped are shown below each gene symbol. ECM, extracellular matrix.
